# How to Choose the Correct Drug in Severe Pediatric Asthma

**DOI:** 10.3389/fped.2022.902168

**Published:** 2022-06-02

**Authors:** Andrew Bush

**Affiliations:** ^1^National Heart and Lung Institute, Imperial College, London, United Kingdom; ^2^Imperial Centre for Paediatrics and Child Health, London, United Kingdom; ^3^Royal Brompton Hospital, London, United Kingdom

**Keywords:** asthma, atopy, eosinophil, immunoglobulin E, Type 2 inflammation, SMART regime, inhaled corticosteroids

## Abstract

When a child with severe asthma (asthma defined clinically for the purposes of this review as wheeze, breathlessness, and chest tightness sometimes with cough) does not respond to treatment, it is important to be sure that an alternative or additional diagnosis is not being missed. In school age children, the next step is a detailed protocolized assessment to determine the nature of the problem, whether within the airway or related to co-morbidities or social/environmental factors, in order to personalize the treatment. For example, those with refractory difficult asthma due to persistent non-adherence may benefit from using budesonide and formoterol combined in a single inhaler [single maintenance and reliever treatment (SMART)] as both a reliever and preventer. For those with steroid-resistant Type 2 airway inflammation, the use of biologicals such as omalizumab and mepolizumab should be considered, but for mepolizumab at least, there is a paucity of pediatric data. Protocols are less well developed in preschool asthma, where steroid insensitive disease is much more common, but the use of two simple measurements, aeroallergen sensitization, and peripheral blood eosinophil count, allows the targeted use of inhaled corticosteroids (ICSs). There is also increasing evidence that chronic airway infection may be important in preschool wheeze, increasing the possibility that targeted antibiotics may be beneficial. Asthma in the first year of life is not driven by Type 2 inflammation, so beyond avoiding prescribing ICSs, no evidence based recommendations can be made. In the future, we urgently need to develop objective biomarkers, especially of risk, so that treatment can be targeted effectively; we need to address the scandal of the lack of data in children compared with adults, precluding making evidence-based therapeutic decisions and move from guiding treatment by phenotypes, which will change as the environment changes, to endotype based therapy.

## Introduction

For the purposes of this review, it will be assumed that the patient has undergone a full diagnostic workup, eliminating as far as possible non-asthma diagnoses, and seeking positive evidence for the diagnosis of asthma (Table 1), acknowledging that there is no one “asthma test” that can definitively diagnose the disease. The protocolized approach to evaluating patients with school age asthma apparently not responding to treatment has been discussed in detail elsewhere (1–4) and will not be described here; the focus is pharmacotherapy, but the importance of social and environmental factors cannot be overstated. Unfortunately, no such protocols have been evaluated in preschool children, although the general principles [check there is no alternative or associated diagnosis, assess adherence objectively (5–7), look for exposure to tobacco and e-cigarettes and allergens, and assess psychosocial factors] will apply.

**TABLE 1:** Diagnostic clues suggestive of another diagnosis, and positive indications that asthma is in fact the diagnosis.

Features suggestive that asthma is not the diagnosis	Positive indicators of an asthma diagnosis
History • Respiratory sounds other than true wheeze • Prominent upper airway disease • Symptoms from first day of life • Sudden onset of symptoms • Chronic wet cough for >4–8 weeks • Continuous, unremitting symptoms • Easy vomiting, heartburn, difficult to feed • Evidence of systemic illness or immunodeficiency	Variable airflow obstruction • Acute response to SABA if reduced FEV_1_ • Positive exercise or other challenge test • Variable spirometry at home with time or after SABA treatment
Physical examination • Clubbing, weight loss, failure to thrive • Upper airway disease tonsillar enlargement, severe rhinitis, nasal polyps • Unusually severe chest deformity • Abnormal, non-wheeze auscultatory signs, e.g., *fixed* monophonic wheeze, stridor, asymmetrical signs • Signs of cardiac or systemic disease	Atopic sensitization • Positive skin prick tests • Positive sIgE
Initial screening tests • CXR: focal changes, excessive airway thickening, and dilatation • FV curve: should be normal or have reduced flows at low lung volumes	Airway inflammation • Elevated FeNO • Raised peripheral blood eosinophil count • Induced sputum eosinophilia

*Obviously, there is no definitive diagnostic test for asthma. CXR, chest radiograph; FeNO, fractional exhaled nitric oxide; FEV_1_, first second forced expired volume; FV, flow volume; SABA, short-acting β-2 agonist; sIgE, specific immunoglobulin E.*

## Key Definitions

Key to personalizing medication in asthma is a clear understanding of what the term means. The *Lancet* commission (8) defines asthma as a clinical syndrome of wheeze, chest tightness, and dyspnea, sometimes with increased cough, and this is the definition used here. This umbrella definition means that, on an individual basis, the underlying cause of the symptoms must be determined, by deconstructing the airway (Table 2) with a particular focus on defining what is treatable (“Treatable traits”) and what treatment success will look like. This is especially important in the preschool asthmas (below).

**TABLE 2:** Deconstructing the airway to plan treatment.

Airway component	Underlying cause	Potential treatment?
Fixed airflow obstruction	1. Developmentally acquired, e.g., maternal smoking in pregnancy 2. Airway remodeling	Avoid worsening obstruction by tobacco, pollution Do not over-treat, trying to reverse the irreversible
Variable airflow obstruction	1. ASM constriction 2. Airway instability/malacia 3. Intraluminal mucus or inflammatory debris	1. SABA, LABA 2. Might need CPAP 1. Airway clearance, mucolytics
Airway inflammation	1. Present or not? 2. Eosinophilic? 3. Neutrophilic? 4. Mixed picture? 5. Beneficial or not?	2. Eosinophilic: ICS, omalizumab, mepolizumab 3. Neutrophilic: consider azithromycin, but look for other diagnoses such as GERD, CF
Airway infection	Bacterial, viral, fungal	Consider targeted antibiotics for any bacterial infection
Augmented cough sensitivity?	Unknown	None licensed in children

*ASM, airway smooth muscle; CF, cystic fibrosis; CPAP, continuous positive airway pressure; GERD, gastro-esophageal reflux disease; ICS, inhaled corticosteroid; LABA, long-acting β-2 agonist; SABA, short-acting β-2 agonist.*

The next definition is, what constitutes severe asthma? Traditionally, this has been defined by the levels of prescribed medication (9, 10) (e.g., Table 3); although confusingly, many different definitions exist (11). However, definition solely by dose and numbers of medications prescribed is not adequate for clinical practice; around half the asthma deaths reviewed in the United Kingdom were not prescribed medications at a “severe” level (12). Risk needs to be incorporated (13, 14), preferably guided by objective biomarkers (15). Risk is multifaceted, and includes risks of side effects of medication and risk of failure of normal airway growth, but particularly, risk of a severe attack. Markers of risk of an attack include a previous severe attack, under-use of inhaled corticosteroid (ICS), over use of short-acting β-2 agonists (SABAs), failure to attend routine asthma checks, and multiple emergency visits for asthma (14). These factors must be considered when choosing treatment. Unfortunately, there are no internationally accepted definitions for preschool children. Empirically, I here define severe preschool asthma as chronic symptoms (most days a week), especially acute attacks of wheeze despite trials of prescribed ICS at doses of 400 μg/day of beclomethasone equivalent and the leukotriene receptor antagonist (LTRA) montelukast.

**TABLE 3:** Definition of severe asthma by levels of medication, modified from Bel et al. (9).

High dose ICS (>500 mcg FP/day age 6–12 and >1,000 age over 12 years, plus LABA, LTRA, and oral theophylline, or failed trials of these add-ons unless clinically contra-indicated, or systemic CS >50% of the previous year required to control asthma, or uncontrolled asthma despite these medications
Uncontrolled asthma (or better, unacceptable asthma outcomes, defined as:
• Chronic symptoms (ACT < 20)
• >2 asthma attacks/year treated with systemic CS
• One serious attack defined as hospitalization, PICU admission, or need for IPPV
• Persistent airflow limitation: FEV_1_ < 1.96 *Z*-scores despite a course of systemic corticosteroids and acute SABA administration
Controlled asthma that worsens on tapering high doses of ICS or systemic CS, or additional biologics

*ACT, asthma control test; CS, corticosteroid; FEV_1_, first second forced expired volume; FP, fluticasone propionate; ICSs, inhaled corticosteroids; IPPV, intermittent positive pressure ventilation; LABA, long-acting β-2 agonist; LTRA, leukotriene receptor antagonist; PICU, pediatric intensive care unit; SABA, short-acting β-2 agonist; Z-score, standard deviation score.*

Importantly, there are different categories of school age severe asthma mandating different approaches (16, 17). Worldwide, the most common is severe asthma due to the unavailability or lack of access to basic medications, either in low- and middle-income settings or in poverty pockets in high-income countries (HICs) (18). This requires political solutions, and is not discussed here. The other categories are difficult asthma (which will be cease to be difficult if basic management is got right); asthma plus co-morbidities; and true severe, therapy-resistant asthma. With energetic multidisciplinary team (MDT) management, difficult asthma and asthma plus may not require additional therapy, but failure to respond puts the patient in the categories refractory difficult asthma or refractory asthma plus, mandating further consideration of pharmacotherapy.

Finally, before embarking on matching patients to prescription, it is worth reflecting on this quotation from Oscar Wilde “To do nothing at all is the most difficult thing in the world, the most difficult and the most intellectual” – and doing nothing (at least in terms of prescribing more medication) may be the most intellectual course. Two studies demonstrate the truth of this maxim in this context. A well-designed study addresses the question as to whether azithromycin or montelukast was the better add-on therapy in symptomatic patients despite ICS and long-acting β-2 agonist (LABA) being prescribed (19). The study ended in futility because most either did not have asthma or were not taking their treatment. Another study of inner city children which aimed to see if the addition of the measurement of fractional exhaled nitric oxide (FeNO) improved asthma outcomes was also futile (20), because during the 2-week run-in period, with detailed attention to the basics of management, asthma control improved out of all recognition and there was virtually no scope for extra benefits during the study. Prescribing nothing extra, but getting the basics right.

## School Age Asthma: Deconstructing the Airway in Pediatric Severe Asthma

The basic components of this process are presented in Table 2. A logical sequence of questions should be asked in order to personalize therapy. For example, it surely makes no sense to give ever more potent anti-eosinophil medications if there is no evidence of airway eosinophilia.

1. Is there fixed airflow obstruction? This is not a treatable trait, but should be identified to prevent over-treatment, trying to reverse the irreversible. There is no agreed protocol to exclude persistent airflow limitation. Spirometry is performed after some form of systemic steroid trial and SABA administration. We use a single intramuscular injection of triamcinolone (40 mg if child <40 kg in weight, otherwise 80 mg) so adherence is assured.
2. Is there specifically SABA responsive variable airflow obstruction? This cannot be determined by acute SABA administration if the child does not have airflow obstruction at the time of examination. However, it can be determined using a challenge test (e.g., exercise or methacholine) or in home using preferably spirometry to ascertain whether there is spontaneous fluctuation in airflow obstruction.
3. Is there evidence of ongoing inflammation, and if so, what is its nature? This is a complex issue in severe asthma.
  – First, whether the (at least potentially) treatable trait of airway eosinophilia is present should be determined. The most direct route is fibreoptic bronchoscopy (FOB) with bronchoalveolar lavage (BAL) and endobronchial biopsy, but this is invasive, and induced sputum is a viable alternative (not in preschool children, below). Peripheral blood eosinophil count certainly correlates with airway eosinophilia (21), but agreement is far from perfect (below) even in the absence of confounders like parasitic disease and non-asthma airway disease.
  – If the phenotype airway eosinophilia is present, what is the endotype? There is usually evidence of Type 2 inflammation with signature cytokines interleukin (IL)-4, -5, and -13, but this is not invariable (below).
  – Is there evidence of activation of other inflammatory pathways, such as IL-17? If there is airway neutrophilia, is this beneficial (response to infection) or adverse (release of neutrophil granule contents leading to tissue damage)? Whereas in adults neutrophilic asthma is refractory to therapy (22, 23), in children at least intraepithelial neutrophils are associated with better asthma outcomes (24).
4. Is there evidence of airway infection? This may be particularly relevant in preschool children (below). If this is the case, targeted antibiotics may be indicated. If the child is prescribed high-dose ICS and there is no evidence of airway eosinophilia, consideration should be given to a dose reduction, given the evidence that ICS may cause clinically significant mucosal immunosuppression (25–28).

## Choosing the Right Medications for School Age Refractory Difficult Asthma

The usual context is the child who is not given or will not take standard medications even despite the MDT intervention. The differential diagnosis is true therapy-resistant asthma, the medications therefore not being given because they are not effective. To resolve this, the next step is to see if there is a response when medication administration is directly supervised, either by an admission to hospital or in the community. If, as is usual, asthma symptoms disappear, FeNO normalizes, and spirometry improves, severe therapy-resistant asthma is excluded and the young person is diagnosed with steroid-sensitive, eosinophilic asthma (5). Ideally directly observed, effective therapy is continued, but often this is not practical. In that event, I would switch the young person to a single maintenance and reliever treatment (SMART) regime, using a combined ICS and LABA (budesonide and formoterol, respectively) inhaler (29, 30). I would ensure that the young person did not have any possibility of accessing SABAs. Of note, GINA recommends this approach at all levels of asthma severity (31), on the basis of ample evidence (32). I would adjust the aspects of the regime (how much regular therapy and the inhaler strength) on an individual basis (Table 4). An additional strategy, not licensed or evidence based, would be to use once daily ICS/LABA preparations such as Relvar (Fluticasone furoate and the LABA vilanterol) with budesonide/formoterol as reliever therapy. Finally, although United Kingdom guidelines insist on ensuring adherence before biologicals can be funded, I take the view that every measure to keep the child from dying from an asthma attack is fully justified (33). A young person must not be penalized because the parents/carers will not ensure ICS is taken regularly and correctly. The hope is that these measures will buy time for increasing age and maturity to bring a new attitude to asthma medications. In any case, good adherence is virtually impossible to confirm in routine clinical practice, although of course non-adherence (e.g., failure to collect prescriptions) is often readily apparent.

**TABLE 4:** Choosing the right medications for school age refractory difficult asthma (when appropriate).

Refractory character	Action
Poor adherence despite every effort to improve	• Check responds to DOTS • Try SMART, Relvar • Confirm eosinophilic airway disease and prescribe biologicals
Ongoing allergen exposure to which child is sensitized, with family unable or unwilling to remedy this	• May be worth trying higher dose ICS, but beware side-effects • Confirm eosinophilic airway disease and prescribe biologicals
Ongoing passive (or active) exposure to cigarettes or vapes despite referral to a smoking cessation clinic	• Phenotype airway to ensure no untreated TH2 inflammation • Consider azithromycin as some adult evidence that smokers asthma may be neutrophilic
Ongoing psychosocial issues	• Ensure symptoms are really due to asthma • Treat any adherence issues (above) • Phenotype airway to ensure all standard treatment is optimal

*DOTS, directly observed therapy; SMART, single maintenance and reliever treatment.*

## Choosing the Right Medications for School Age Refractory Asthma Plus Comorbidities

### Obesity Asthma With Failure of Weight Reduction

This is a situation that requires very careful evaluation. The definition of severe asthma includes chronic symptoms, but it is essential to be sure that these are actually due to asthma. Exercise intolerance due to obesity and deconditioning will not respond to intensifying asthma therapy. This is a general problem – in one big epidemiological study, around half of young people complaining of shortness of breath on exercise had neither exercise-induced bronchoconstriction nor exercise-induced laryngeal obstruction, despite which many had been treated with inhaled therapy for asthma (34). It is, therefore, of primary importance to determine whether symptoms are truly due to asthma before prescribing.

– Fixed airflow obstruction: common but not exclusive to obese young people is dysanaptic airway growth, defined as a normal first second forced expired volume (FEV_1_), a greater than normal forced vital capacity (FVC), and therefore a reduced FEV_1_/FVC ratio (35). The exact determinants are unclear, but murine studies implicate antenatal nicotine exposure (36), and in humans, excessive weight gain in the first 2 years of life, irrespective of birth weight (37). Dysanaptic growth is associated with worse asthma outcomes, but is not amenable to current therapies.
– Variable airflow obstruction: Related to the need to determine the exact cause of symptoms is the need to be sure that variable airflow obstruction is SABA-responsive, and not due to variable atelectasis related to reduced chest wall compliance. Objective documentation, preferably with spirometry, is essential.
– Airway inflammation: Obesity does not protect against atopic allergic inflammation (38), and obese patients may need escalation of therapies addressing this issue. However, the presence of Type 2 inflammation must first be documented, not only because excessive steroid therapy may worsen obesity, but also because alternative inflammatory pathways may play a role in obesity. Obesity is well known to be a systemic, pro-inflammatory state, and there is evidence that some obese asthma is driven by systemically released IL-6 targeting the airways independent of immunoglobulin E (IgE) or blood eosinophil levels. The advent of the coronavirus disease 2019 (COVID-19) pandemic has led to two licensed approaches to treatment targeting IL-6 in this context. Siltuximab is a monoclonal antibody that binds to IL-6 (39), and sarilumab (40) and tocilizumab (41) are monoclonal antibodies that bind to the IL-6 receptor. Some of these approaches have been used in other contexts, for example, tocilizumab in interstitial lung disease (42), but as yet not in asthma according to the best of my knowledge. However, this might be a therapy for systemic IL-6-driven asthma in the future.

In summary, the breathless obese young person poses particular challenges, and it is essential to measure pathology and personalize therapy rather than blindly prescribing ever more therapies.

### Severe Rhinosinusitis

The relationship between upper and lower airway disease has long been debated, but there is compelling evidence that treatment of severe rhinosinusitis can improve asthma control, and treating the upper airway with nasal steroids and anti-histamines may be helpful before escalating asthma therapy (43). It should be noted that nasal steroids may, however, significantly contribute to adrenal suppression (44).

### Breathing Pattern Disorders

There is a spectrum of these including hyperventilation syndromes and exercise-induced laryngeal obstruction (45). Their importance is related to the fact that they are frequently mis-diagnosed as uncontrolled asthma and treatment escalated. Detailed evaluations by specialist respiratory physiotherapists, speech and language therapists, and clinical psychologists, combined with cardiopulmonary exercise testing while laryngoscopy is performed, may help with the diagnosis and guide therapy. Again, escalation of asthma medications is not helpful.

The various options are summarized in Table 5.

**TABLE 5:** Choosing the right medications for school age refractory asthma plus comorbidities (when appropriate).

Co-morbidity	Action
Obesity with failed weight loss	Confirm symptoms are due to asthma not deconditioning Consider bariatric surgery Determine whether Type 2 inflammation is present and treat accordingly (Future option?) anti-IL-6 strategies if systemic inflammation is the issue
EILO	Reduce medications until signs of Type 2 inflammation (re)appear Involve skilled physiotherapist, speech therapist, and sports psychologist Consider ENT referral if remains refractory
Allergic rhinosinusitis	Identify and avoid the relevant antigens where possible Topical corticosteroids ENT referral for consideration of surgery if refractory symptoms persist despite medical treatment
Food allergy	A non-causative association of worse outcomes, so a marker of risk, make sure airway Type 2 inflammation is well controlled
Gastro-esophageal reflux	Even if symptomatic, treatment makes no difference to asthma outcomes, so only take action if there are disabling symptoms meriting therapy

*ENT, ear, nose, and throat; EILO, exercise-induced laryngeal obstruction.*

## Choosing the Right Medications for School Age Severe, Therapy-Resistant Asthma

The days of prolonged daily or alternate day corticosteroids have fortunately gone. Although an increasing range of monoclonals is becoming available to adult chest physicians, the only current pediatric options are the monoclonals omalizumab, which binds to IgE, and mepolizumab, which binds to IL-5. Dupilumab blocks the receptors for IL-4 and IL-13. The recent Voyager study, which demonstrated the efficacy of dupilumab in 6–11 year olds (below), raises hopes that this too will become available to pediatricians (46). The biologicals have to be given *via* injection every 2–4 weeks. We now have a program whereby this can be done at home by direct videolink (47).

### Omalizumab

This monoclonal complexes with IgE preventing it from binding to the high-affinity IgE receptor (FceRI) on mast cells and basophils which would lead to mediator release. There is, by far, the most pediatric experience with this monoclonal. Dosage depends on the body weight and IgE level. If total IgE is >1,300 kIU/L (or <75), the medication cannot be used in the United Kingdom, which is important because many severe asthmatics have levels well above the therapeutic range. However, there are international variations in the levels of IgE for which omalizumab may be used, and national guidelines need to be checked. Although in many countries aeroallergen sensitization is a requirement for funding, this is illogical because in adults who are not sensitized but have a high IgE the results of treatment are not inferior. The Cochrane review (48) demonstrated a reduction in asthma attacks: [odds ratio (OR) 0.55, 95% confidence interval (CI) 0.42–0.60 in 10 studies recruiting 3,261 patients] with an absolute reduction of 26–16%; reduced hospitalizations (OR 0.16, 95% CI 0.06–0.42; 4 studies that recruited 1,824 patients) with an absolute reduction 3–0.5%; reduced SABA usage (OR 0.16, 95% CI 0.06–0.42; 4 studies that contained 1,824 participants); absolute small but significant reduction in SABA (mean difference −0.39 puffs per day, 95% CI −0.55 to −0.24; 9 studies, 3,524 patients). A systematic review of real-life efficacy in adults and children summarized 86 manuscripts. Treatment effectiveness was excellent or good in 77% patients at 16 weeks (95% CI 0.70–0.84) and in 82% at a year (0.82, 0.73–0.91). The improvements in FEV_1_ and Asthma Control Questionnaire (ACQ) were small. The greatest benefits were in the reduction of severe attacks [risk ratio (RR): 0.41, 95% CI: 0.30–0.56], patients receiving oral corticosteroids (RR: 0.59, 95% CI: 0.47–0.75), and number of unscheduled physician visits (mean difference: −2.34, 95% CI: −3.54 to −1.13) in the first year of treatment (49). A French study (50) reported data in 101 children, of whom 92 were still receiving treatment after a year (6 discontinued due to severe adverse effects). Severe asthma attack rate and hospital admissions dropped dramatically (72%, from 4.4 per patient during the preceding year to 1.25 during the treatment year, and 88.5%, 44% during the preceding year to 6.7% during the treatment year, respectively). There was also an improvement in asthma control (0% at baseline to 67% well-controlled at 1 year); a 30% decrease in ICS dose (baseline 703–488 μg fluticasone equivalent/day at a year) but unsurprisingly only a small increase in FEV_1_ (88–92.1% predicted). At 2 years (51), 73 (79.3%) were still receiving the treatment. Treatment had been discontinued in further 15 patients due to the lack of improvement (*n* = 4), adverse events (*n* = 8), lost to follow-up (*n* = 4), and personal reasons (*n* = 3). Severe attacks decreased to a mean (95% CI) of 0.22 (0.03–0.41) per year. No patient needed to be hospitalized. Level of control, spirometry, and daily ICS dose did not change significantly. Taken together, these data show a sustained benefit for omalizumab, in particular, in the reduction of severe attacks. An updated systematic review in children is awaited (52).

To select suitable children for omalizumab therapy remains unclear. Levels of total (53, 54) or specific IgE (sIgE) (55) are not reliable predictors. A study was performed in 850 patients of age 12 and over related the reduction in asthma attacks over a 48 week period to levels of FeNO (*n* = 394), blood eosinophils (*n* = 797), and serum periostin (*n* = 534) (56). Attack reduction was greater in the high subgroups for FeNO and blood eosinophils vs. placebo, respectively, 53% [95% CI, 37–70 vs. 16% (95% CI, 34)] to 46) and 32% (95% CI, 11–48 vs. 9% (95% CI, −24 to 34). Periostin levels showed no statistically significant effect (and in any event, since periostin is released from growing bone, it is not a useful pediatric biomarker). These data suggest that (a) T-helper (TH)2 high adults with multiple asthma attacks will have the best response; and (b) by analogy, these will be predictive biomarkers in children. However, this needs to be tested, and there are also problems with using adult blood eosinophil cutoffs in children (below).

### Mepolizumab

There is convincing evidence for the efficacy and safety of mepolizumab in young people of age 12 years and over and in adults (57). A blood eosinophil count of >300 cells/μl is a good biomarker of efficacy (58). However, less than 100 patients aged less than 16 have been included in these studies. There are some limited pediatric data that show safety and a reduction in blood eosinophil count with mepolizumab (59, 60), but there are no large-scale efficacy data, despite which mepolizumab has been licensed for use in children.

### Dupilumab

There is extensive evidence for efficacy and safety of dupilumab in the treatment of children and adults with eczema (61) and in children 12 years and over and in adults with asthma (62), but until recently, no evidence of efficacy has been observed in school age asthma. The Voyager study (46) recruited 408 children of age 6–11 years with uncontrolled moderate-to-severe asthma. At baseline, children were required to have either a TH2 inflammatory asthma phenotype (≥150 blood eosinophils per cubic millimeter or FeNO of ≥20 ppb) or a blood eosinophil count >300 cells/μl. In the TH2 inflammation group, severe asthma attacks were reduced by dupilumab [0.31 (95% CI 0.22–0.42) vs. placebo 0.75 (95% CI, 0.54–1.03) (relative risk reduction by dupilumab, 59.3%; 95% CI, 39.5–72.6; *P* < 0.001)]. There was a small but significant improvement in FEV_1_ of 10.5 ± 1.0% with dupilumab compared with placebo (5.3 ± 1.4, *P* < 0.001) and better asthma control (*P* < 0.001). The results were similar in those with a baseline eosinophil count >300 cells. The medication was safe and well tolerated.

### Specific Issues With Selecting TH2 Inflammation Strategies in Children

These are (a) the biology of severe asthma in children; (b) the use of eosinophils as a biomarker; and (c) the developmental role of the eosinophil in children, which last may lead to safety questions specific to the pediatric age group.

In the pediatric literature, by no means all severe asthma appears to be driven by TH2 inflammation. We phenotyped a large group of children with severe asthma who had been through our protocol for the assessment of severe asthma. Many, but not all, were eosinophilic on induced sputum, BAL, and endobronchial biopsy, but evidence of TH2 inflammation was scant in all three compartments (63). The mechanisms of eosinophilia in this group have not been determined, but non-TH2 eosinophilia has been described in other contexts (64). The US Severe Asthma Research Program (SARP) network (65) reported 53 children with asthma of whom 31 were severe, and 30 adult controls. They found that the best discriminants between asthma and controls were BAL IL-6 and IL-13. Severe asthma was differentiated from moderate disease by CXCLI, growth related oncogene (GRO), regulated on activation, normal T expressed and secreted (RANTES, CCL5), IL-12, interferon (IFN)-γ and IL-10. When alveolar macrophage lysate was studied, IL-6 was the best discriminant. They concluded that severe asthma in children was not characterized by either a TH1 or TH2 signature. A further study (66) utilizing *n* = 68 BAL from 52 children with severe, therapy-resistant asthma showed that viruses and bacteria were commonly detected. Although CCR5 positive TH1 cells were enriched in BAL, there were also pro-inflammatory, TH1, TH17, and TH2 profiles detected; of note, there was no control group. Further findings were that TH2 skewing correlated with total serum IgE. Those who were multi-sensitized showed increased IL-5, IL-33, and IL-28A/IFN-λ2. Not all sIgEs had equivalent effects; changes correlated with sIgE to house dust mite, ryegrass, and fungi but not with sIgE to cats, ragweed, and food allergens, which is another important confirmation that atopy is not an “all-or-none” state (67, 68). Only BAL IL-5 increased with age and correlated with BAL and blood eosinophils. Of course, in all these cross-sectional studies, causation cannot be inferred from correlation. Also, when considering treatment the question should be “does it work?” rather than “should it work?,” but it is clear that severe asthma has multiple endotypes and this needs to be factored into decisions about trials of treatments. The recommendation would be therefore to clearly define that the disease is truly TH2-driven in a given individual, including if necessary proceeding to bronchoscopy.

A global perspective is also important. Most of the invasive studies come from HICs, and it should not be assumed that severe asthma is the same in low- and middle-income countries (LMICs). It would be a mistake uncritically to follow HIC protocols in LMIC settings.

Blood eosinophil count is a hallowed marker for airway eosinophilia in adult asthma (57) and chronic obstructive pulmonary disease (69), but there is a problem. In children, the normal blood eosinophil count is much higher than in adults, dropping to adult levels throughout childhood (70). Even in adult life, asthmatics with a normal blood eosinophil count may respond to Type 2 biologics (58). This suggests that adult blood eosinophil levels may not be appropriate in guiding decisions in children, but perhaps also, there may be patients (adults and children) with low blood eosinophils who may yet have airway eosinophilia, and additional markers of this are needed. Furthermore, in LMICs in particular, where there is a high parasite burden, “normal” blood eosinophil count may be even higher. The ideal would be to use at least induced sputum to confirm directly that airway eosinophilia is present before instituting Type 2 biologics.

Finally, the assumption that the eosinophil has no beneficial effects needs to be challenged. Even in adults, it would seem that too aggressive an obliteration of circulating eosinophils may be adverse. Benralizumab leads to a much more dramatic reduction in circulating eosinophils then mepolizumab and reslizumab, but is associated with more respiratory infections and more infection-driven asthma attacks (71). A number of studies have attributed important homeostatic functions to the eosinophil, at least in animal models. These include Beige fat thermogenesis and glucose homeostasis; adjuvant-induced B-cell priming and maintenance of memory plasma cells; antigen presentation in the intestine (72–75). Additionally, eosinophils have antiviral properties (76). In an observational study, asthmatic adults infected with COVID-19 were less likely to be admitted, and less likely to die, if they had a high blood eosinophil count (77). This is not to decry the value of anti-eosinophil strategies, merely to highlight that the risk benefit equation may be different in children.

## Choosing the Right Medications for Severe Preschool Asthma

Asthma in preschool children is also defined clinically as above; the question?; “at what age can asthma be diagnosed?” is without meaning (78); in the preschool years, attempts should be made to deconstruct the airway prior to escalating treatment exactly as in school age, although this may be more difficult to achieve. Historically, all preschool wheezers were lumped together and treated identically. In 2008, an ERS, 2008 guideline formalized the distinction between episodic viral wheeze (EVW, wheeze solely with a usually clinically diagnosed viral respiratory tract infection), and multiple trigger wheeze (MTW), in which there are symptoms with typical asthma triggers such as exercise even in between viral infections (79). Given that early administration of ICS does not prevent school age asthma developing (80–82), the recommendation was that EVW should be treated intermittently, with ICS or LTRAs montelukast), and MTW with regular ICS. It was recognized that these symptom-based phenotypes could change over time. In a subsequent iteration (83), it was suggested that really severe EVW merited a trial of regular ICS. However, it became clear that the agreement between underlying pathological phenotypes and symptom patterns was very poor, and furthermore, parental perception of the presence or absence of interval symptoms was frequently unreliable.

Traditionally, the interval between babyhood (when lung function can be performed under sedation) and school age (where active cooperation can be obtained) has been a black hole wherein measurements of pulmonary function cannot be made. However, it is clear that quite young children can be shown how to perform good quality spirometry (84), and bronchodilator responsiveness measured. Another potentially useful technique is forced oscillation. There is no generally accepted definition of persistent airflow limitation, or fixed airflow obstruction, in preschoolers. Current practice (which is not evidence-based) is not to perform a trial of oral corticosteroids, unlike in school age children, but relies on the value obtained after SABA and perhaps inhaled anti-cholinergic administration.

The first real attempt to personalize medicine in preschool wheeze was the INFANT study (85). Preschool wheezing children were given in random order as follows: regular ICS, regulart LTRA, and intermittent ICS. Prespecified subgroups were atopic sensitization, gender, and acute attacks of wheeze, and *post hoc*; blood eosinophil count was added in to the data analyses. In summary, the combination of aeroallergen sensitization and a blood eosinophil count >300 cells/μl predicted a group which was responsive to ICS; in the other patients, it did not matter what treatment was given (there was no placebo group). Of the original 300 patients, 60 improved spontaneously but only 64 were ICS responders, leaving a big unmet need.

A subsequent study has highlighted a potential role of bacterial infection in non-atopic preschool wheeze. A total of 35 children with severe preschool wheeze (*n* = 21 MTW, *n* = 14 EVW, classified clinically with a wheeze video questionnaire) underwent venepuncture for blood eosinophils and total and sIgE, and a clinically indicated FOB, BAL, and endobronchial biopsy with the viral polymerase chain reaction (PCR), bacterial culture, and 16S of amplicon sequencing at a time of clinical stability (86). Notably, 60% had either a positive bacterial culture or viral detection, and 26% had both. The most common bacteria were *Streptococcus pneumoniae*, *Moraxella catarrhalis*, *Staphylococcus aureus*, and *Haemophilus influenzae*, and the most common viruses were rhinovirus, bocavirus, and adenovirus. Unsupervised analysis revealed two bacterial profiles, i.e., a mixed group (*Streptococcus*, *Prevotella*, *Neisseria*, and *Porphyromonas*) and a *Moraxella* group. The latter had increased BAL but not blood neutrophil counts. There was no difference in clinical wheeze phenotype (EVW, MTW) or atopic status between the two groups. There was evidence to suggest bacterial dysbiosis in the *Moraxella* cluster. Subsequently, the same group attempted a cluster analysis of a large group of severe preschool wheezers (87). A total of 136 children aged 1–5 years (105 with recurrent severe wheeze and 31 non-wheezing respiratory disorders) were studied. Treatment was recorded and the following investigations performed: peripheral blood: leukocyte counts, and sIgE to common inhalant and food allergens, allergic sensitization being defined as sIgE ≥0.35 kUA/L to at least one allergen tested; bronchoscopy, BAL, and endobronchial biopsy with bacterial culture and a multiplex PCR to 20 viruses and *Mycoplasma pneumoniae*. Analysis was performed using the partition around medoid (PAM) algorithm coupled with Gower’s distance for mixed data and eight variables were used to determine the clusters. These were blood and BAL neutrophil and eosinophil counts, atopic status, a positive viral PCR and bacterial culture, and prescription of ICS. Interestingly, BAL eosinophils and peripheral blood neutrophils did not distinguish between the clusters. Of the severe wheezers, 30/105 were classified as EVW and 44/105 as MTW; in 28, or more than a quarter, it was unclear in which category they belonged, further calling into question the utility of history taking to guide therapy. There were four clusters determined, which bore no relation to clinical wheeze phenotypes. In cluster 1 (24/134, 17.9%), all were sensitized, and there were the highest blood eosinophil counts [mean = 5.54%, standard deviation (SD) = 2.86%], highest ICS doses use (91.7%), and a moderate rate of bacterial (69.5%, especially *Moraxella*) and viral detection (56.5%). In cluster 2 (42/134, 31.3%) there were low BAL neutrophils (mean = 9.44%, SD = 13.89%), and a low rate of positive bacteriology (17.1%) and viral detection (15.0%). All had been prescribed ICS. In cluster 3 (*N* = 31/134, 23.1%) there was the highest rate of bacterial (*H. influenzae*, *S. aureus*, and *Streptococci*) and viral infection (96.8 and 86.7%, respectively), associated with the highest BAL neutrophil counts (mean = 31.7%, SD = 25.11%); 67.7% had been prescribed ICS. Finally, in cluster 4 (*N* = 37/134, 27.6%): no patient was prescribed ICS, most were non-atopic, and the most prominent symptom was persistent cough but not wheeze. Possible treatment implications are given in Table 6.

**TABLE 6:** Clusters of preschool wheeze, and possible implications for treatment (85).

Cluster number	Clinical features	Potential treatment
Cluster 1	100% sensitized, highest blood eosinophils (mean = 5.54%, SD = 2.86%), high ICS use (91.7%), and moderate rate of bacterial (69.5%, especially *Moraxella*) and viral detection (56.5%)	Highly atopic and eosinophilic ICS or even consider Type 2 biologics (unlicensed in most countries)
Cluster 2	Low BAL neutrophils (mean = 9.44%, SD = 13.89%), low rate of positive bacteriology (17.1%), and viral detection (15.0%). All has been prescribed ICS	Low BAL neutrophils and low infection burden Consider LAMA
Cluster 3	Highest rate of bacterial (*HI*, *SA*, *streptococcal*) and viral infection (96.8 and 86.7%, respectively), and the highest BAL neutrophils (mean = 31.7%, SD = 25.11%); 67.7% had been prescribed ICS	No atopy, high infection burden Targeted antibiotics
Cluster 4	Mostly non-atopic with cough the main symptom, but note that 20% of severe wheezers were in this cluster	No sensitization, infection, or inflammation Consider LAMA

*BAL, bronchoalveolar lavage; HI, Haemophilus influenzae; ICSs, inhaled corticosteroids; LAMAs, long acting muscarinic agents; SA, Staphylococcus aureus; SD, standard deviation.*

In summary, there is now an evidence base for a subgroup of preschool wheezers (atopic, eosinophilic) to guide treatment, but ideally this needs to be confirmed in a second cohort prospectively. In terms of the infected group, further study is needed. In a small proof of concept trial (88), 60 children aged 1–5 years with ≥2 wheeze attacks in the previous year were categorized as EVW or MTW. The intervention group was prescribed ICS if blood eosinophils ≥3%, or targeted antibiotics if there was a positive culture on induced sputum or cough swab. The control group received standard care. Again, there was no relationship between symptom-based phenotypes and blood eosinophils, atopic status, or infection. Rates of ICS prescription were the same (67%), around half had an unscheduled health care visit, and time to unscheduled visit was the same. Each group were prescribed ICS. There were no differences in any parameter between those who did and did not have an UHCV. Blood eosinophil-driven ICS treatment did not impact outcomes, but ICS adherence was poor. Clearly, until adherence is addressed and there is buy-in to the concept of stopping ICS in the non-allergy, low eosinophil group, it will be difficult to progress these concepts.

## Choosing the Right Medications for Severe Asthma in the First Year of Life

First year wheeze is common, but poorly understood (79). We know that even wheeze severe enough to be investigated in a tertiary hospital, even those with atopic sensitization and acute reversibility of airflow obstruction to SABA, is characterized by the absence of Type 2 inflammation (89), so ICS are highly unlikely to be useful. Understanding first year wheeze is a major research priority for the future. At the moment, all we can offer is trial and error of bronchodilators and possibly LTRA.

## The Future: Where Are We, and Where Do We Need to Go?

**FIGURE 1:**
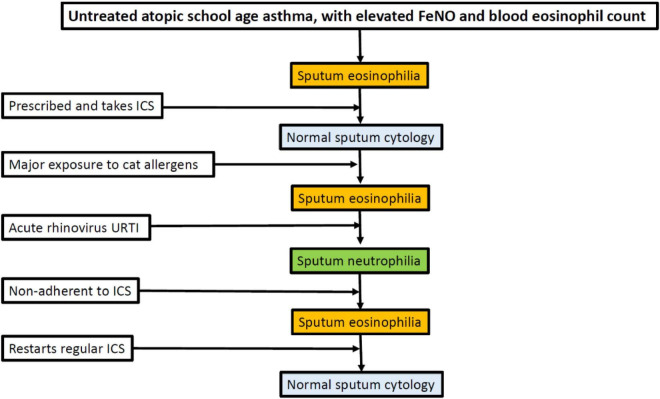
Potential variability of sputum phenotype in an individual who is cat allergic and has asthma. ICSs, inhaled corticosteroids; FeNO, fractional exhaled nitric oxide; URTI, upper respiratory tract infection.

Six important areas of unmet need are as follows:

1. *Measurement in clinical practice: For too long, we have been contented with asking questions and chest auscultation without making objective measurements. This is plain wrong in the 21st century. We need a measurement culture in the respiratory clinic. The fact the tools may be difficult to use is not an excuse to discard them when planning treatment. Physiological measurements can be made, and skin prick tests easily performed, and blood eosinophils are now a point-of-care test. We must not let inertia lead to discrimination against young children or be contented with a lower standard of care in this group compared with adults and school age children.*
2. *Research in children: It is an absolute disgrace that there are huge evidence gaps in children. Obvious examples are the use of ICS/LABA as reliever instead of SABA in children under age 12, and, with the honorable exception of VOYAGER, the pitiful lack of efficacy data for most biologicals in children. Clinical trial data in preschool children are even more scant. Legislation is urgently needed to achieve this. The example of cystic fibrosis (CF), in which disease novel small molecule therapy is rapidly accelerated down the age ranges from over 12 years to young babies puts the asthma community to shame.*
3. *Comparison studies: Even with the limited biologics available in school age asthma (omalizumab and mepolizumab), we have no studies comparing the two and are left making haphazard, *N*-of-1 treatment trials. Hopefully, the TREAT trial will address this (90).*
4. *Phenotype stability: We currently try to use phenotype-based therapy (e.g., treatment of airway eosinophilia) but such limited data that are extant show that, for example, cellular phenotypes in sputum are not stable over time, either in severe or moderate asthma (91). This is unsurprising; a phenotype results from the interactions of an organism with its environment, and if the environment changes then so may the phenotype (Figure 1). We are lacking in data on the stability of preschool phenotypes, and validation in a second cohort. As with “asthma genes” a single cohort study cannot be definitive.*

5. *The need to move to determining endotypes: What is really needed, and a destination which is a long way away, is determining the underlying molecular and cellular pathways, which will be robust by definition. This will become more pressing as more biologicals become available. We will need to select medications on the basis of endotypes rather than randomly.*
6. *Biomarkers are desperately needed: If we are to be objective in therapeutic decisions, we need objective biomarkers. This includes biomarkers for risk, in particular risk of a severe asthma attack, so that management efforts including treatment can be focused on those that need it. We also need biomarkers of efficacy, particularly for biologicals. This would enable us to target the right biomarker to the right child and also to do efficacy studies in younger children who may struggle with convantional end-points. Again, the example of CF should be borne in mind. Reduction in sweat chloride by the new molecular therapies is accepted as evidence of efficacy in young children (92), who are so well that demonstrating efficacy by conventional testing would take huge numbers for many years. The other example is the use of *in vitro* testing of novel therapies using cells harvested by nasal brushing or the generation of rectal organoids has been shown to correlate with *in vivo* treatment response (93, 94).*

In summary, we have made considerable progress in objectively choosing therapies for children who are struggling with bad asthma, but we have a long way to go. It is essential that we are not complacent, but ensure that we recognize the length of the journey ahead, and are determined to reach the end, whereby children of all ages are treated on the basis of objectively determined need and response. The last century history and physical examination are simply not adequate or acceptable in the 21st century.

## Author Contributions

The author confirms being the sole contributor of this work and has approved it for publication.

## Conflict of Interest

The author declares that the research was conducted in the absence of any commercial or financial relationships that could be construed as a potential conflict of interest.

## Publisher’s Note

All claims expressed in this article are solely those of the authors and do not necessarily represent those of their affiliated organizations, or those of the publisher, the editors and the reviewers. Any product that may be evaluated in this article, or claim that may be made by its manufacturer, is not guaranteed or endorsed by the publisher.
